# Evolving treatment strategies for HER2-altered non–small-cell lung cancer: the rise of TKIs and ADCs

**DOI:** 10.3389/fonc.2026.1771869

**Published:** 2026-03-25

**Authors:** Sevinc Balli, Tugba Basoglu, Mustafa Ozdogan

**Affiliations:** 1 Department of Medical Oncology, Samsun City Hospital, Samsun, Türkiye; 2 Department of Medical Oncology, Memorial Göztepe Cancer Center, Istanbul, Türkiye; 3 Department of Medical Oncology, Antalya Memorial Hospital, Antalya, Türkiye

**Keywords:** antibody–drug conjugate, brain metastases, HER2-mutant NSCLC, resistance mechanisms, targeted therapy, tyrosine kinase inhibitor

## Abstract

Human epidermal growth factor receptor 2 (HER2) is an actionable oncogenic driver in a small subset of non-small cell lung cancer (NSCLC), occurring as gene mutations and less frequently as gene amplification or protein overexpression. Until recently, HER2-driven NSCLC lacked effective targeted treatments, and patients were managed with conventional chemotherapy and immunotherapy. However, the therapeutic landscape has rapidly evolved with the advent of novel HER2-directed antibody–drug conjugates (ADCs) and tyrosine kinase inhibitors (TKIs). Following the 2022 approval of the antibody–drug conjugate fam-trastuzumab deruxtecan (T-DXd), the therapeutic landscape of HER2-mutant NSCLC further expanded in 2025 with the regulatory approval of two highly selective oral HER2 tyrosine kinase inhibitors, zongertinib and sevabertinib. This review provides a comprehensive update on these emerging HER2-targeted therapies, including their clinical trial data, mechanisms of action, and comparative benefits over older pan-HER agents. We discuss the emerging therapeutic scope in HER2-”low” NSCLC, challenges with immunotherapy in HER2-driven tumors, and known resistance mechanisms to TKIs and ADCs. An evidence-based treatment algorithm is proposed, integrating new agents into current practice and addressing special considerations such as central nervous system (CNS) metastases. We also outline ongoing trials that may further shift first-line standards. In summary, recent breakthroughs in HER2-targeted TKIs and ADCs are transforming outcomes in this rare NSCLC subset, though optimizing sequencing, managing resistance, and improving patient selection through biomarker development remain priorities for future research.

## 1 Introduction

According to GLOBOCAN 2022 data, lung cancer remains the leading cause of cancer-related mortality worldwide, with non-small-cell lung cancer (NSCLC) comprising about 85% of all cases (1, 2). Advances in our understanding of disease biology and the identification of oncogenic driver alterations have profoundly reshaped therapeutic strategies over the past decade (3). Mutations once considered non-targetable—such as KRAS and HER2—are now recognized as actionable targets (4, 5). Consequently, comprehensive molecular assessment is now standard in NSCLC management.

**Figure 1:**
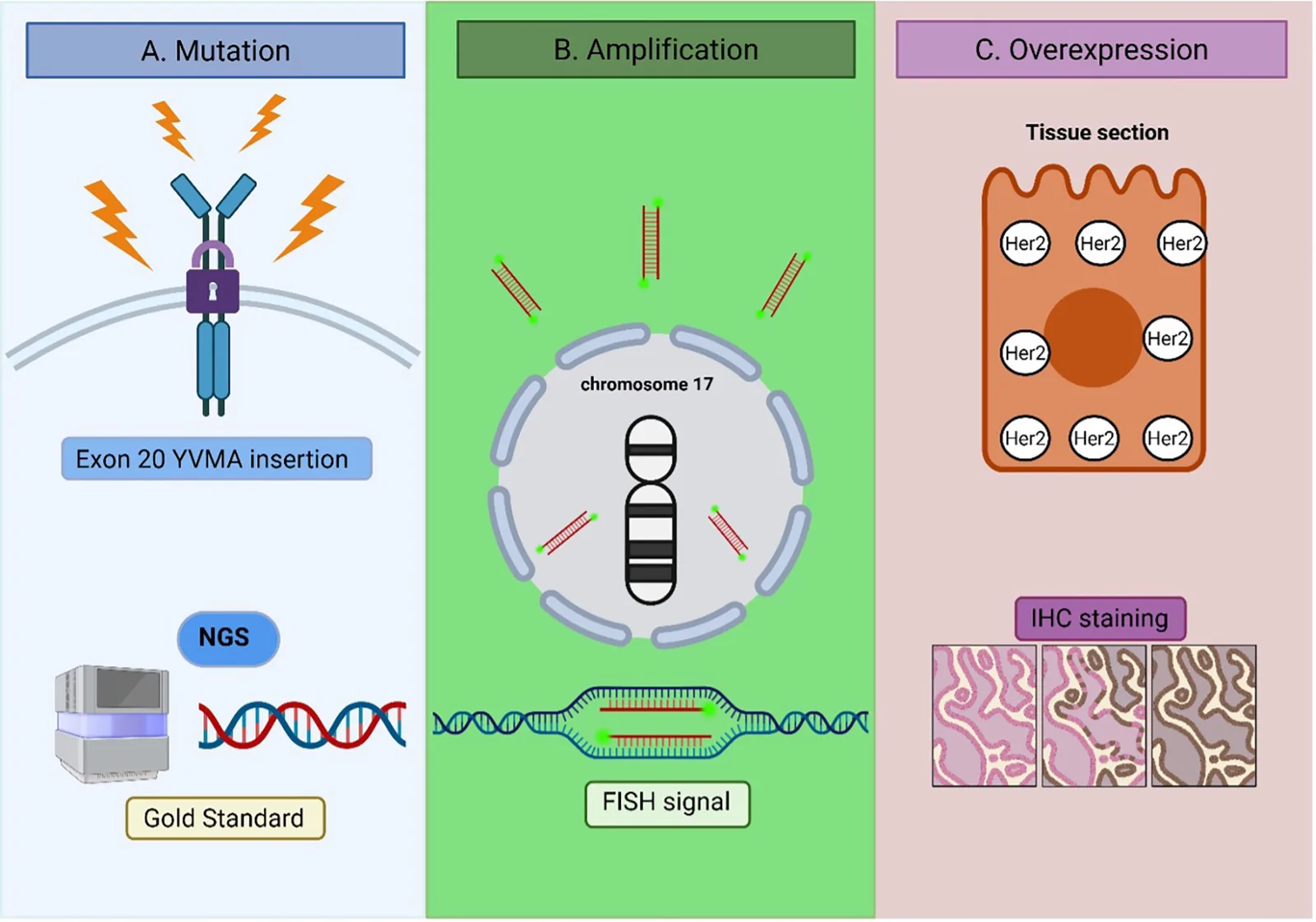
HER2 activation mechanisms and diagnostic landscape.

**Figure 2:**
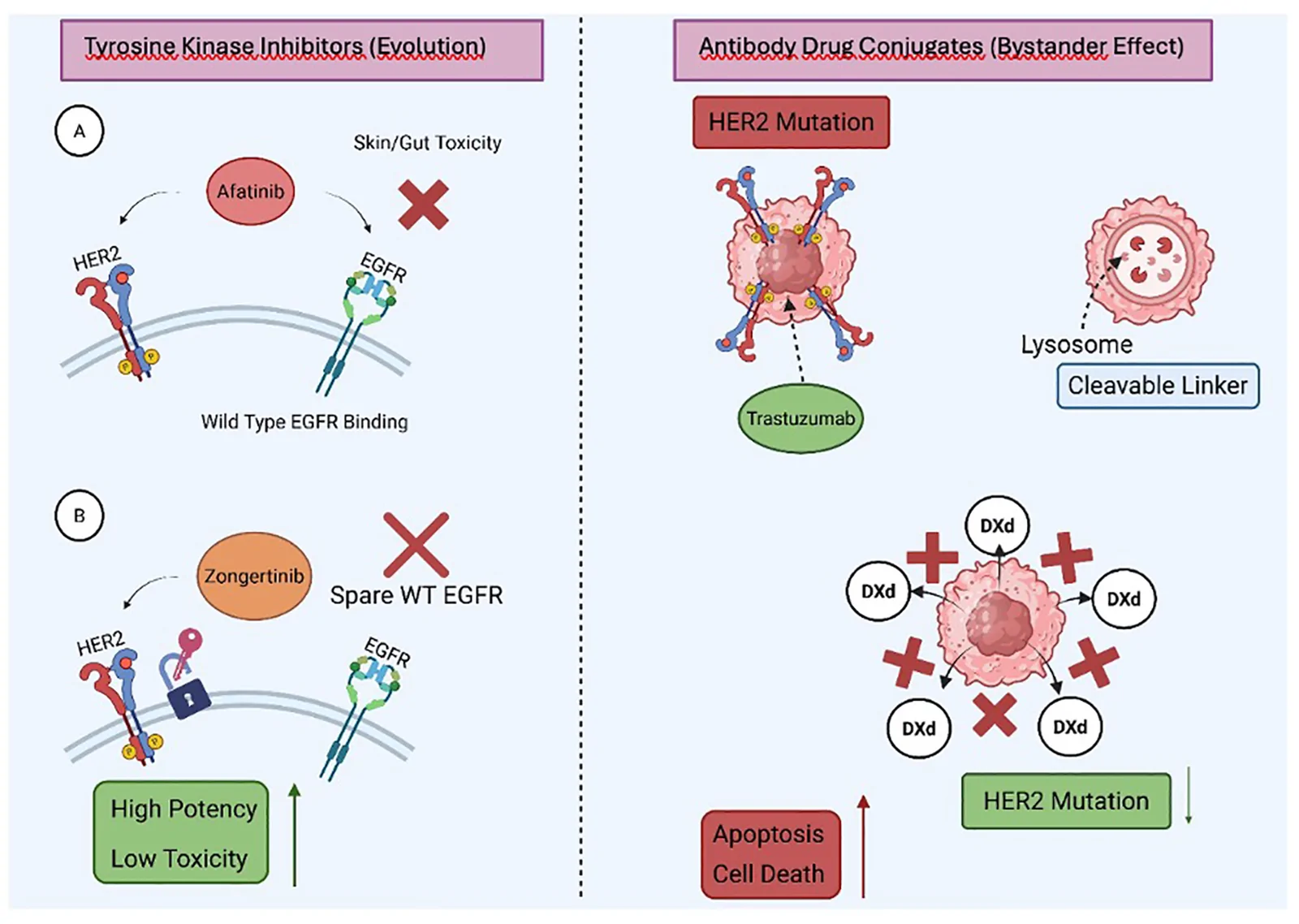
Mechanism of action of next-generation agents: the difference between selective TKI and ADC.

Among these molecular drivers, the human epidermal growth factor receptor 2 (HER2; also known as ERBB2) has emerged as a particularly promising therapeutic target within the ERBB family of receptor tyrosine kinases (6). The ERBB family comprises EGFR (ERBB1), HER2 (ERBB2), HER3 (ERBB3), and HER4 (ERBB4). Unlike the other members, HER2 has no known ligand (**Figure 1**). Ligand binding to the extracellular domain forms catalytically active homo- and heterodimers, which activate downstream pathways that regulate cellular proliferation, differentiation, migration, and apoptosis. In contrast, HER2 exists in a constitutively active conformation, driving persistent signaling through the phosphoinositide 3-kinase–AKT (PI3K–AKT) and mitogen-activated protein kinase–extracellular signal-regulated kinase (MEK–ERK) pathways (**Figure 2**) (6–8).

In NSCLC, HER2 oncogenic activation can occur via mutations as well as via gene amplification or protein overexpression (9). These categories often overlap and frequently co-exist with other genomic alterations, complicating disease characterization. Clinically, HER2-driven NSCLC is more prevalent in younger, never-smoker patients and often presents with an aggressive course, including a high propensity for brain metastases. Historically, treatment outcomes for HER2-altered NSCLC were poor because no HER2-specific therapies were approved. Early clinical investigations in HER2-altered NSCLC relied either on repurposing breast cancer–derived HER2-targeted monoclonal antibodies (such as trastuzumab) or on testing early-generation, non-selective EGFR family TKIs (including afatinib and neratinib), which were originally developed for EGFR-mutant lung cancer and evaluated in HER2-driven disease due to the shared ERBB receptor biology. Overall, these strategies resulted in modest clinical activity (ORR ≤20%) and considerable off-target toxicity (10). Until 2021, the standard of care for advanced HER2-mutant NSCLC remained platinum-based chemotherapy often combined with immunotherapy, as targeted options were lacking. This status quo began to change with the development of ADCs and TKIs specifically targeting HER2 alterations in lung cancer. Trastuzumab deruxtecan (T-DXd) became the first agent approved in 2022 for metastatic HER2-mutant NSCLC, after demonstrating robust efficacy in a phase II trial (11). Since then, multiple next-generation HER2 inhibitors have emerged, propelling HER2-positive NSCLC into an era of precision therapy.

This review summarizes recent advances in HER2-targeted therapy for NSCLC, with a particular focus on the clinical development of next-generation HER2-selective tyrosine kinase inhibitors and antibody–drug conjugates. We critically review the available efficacy and safety data of these agents and discuss how they are reshaping treatment paradigms for HER2-mutant disease. In parallel, we address several evolving and clinically relevant considerations, including the exploratory concept of “HER2-low” expression in NSCLC, the generally limited benefit of immune checkpoint inhibition in HER2-driven tumors, and emerging mechanisms of resistance that may constrain the durability of targeted responses. Finally, we propose an evidence-based treatment sequencing framework that integrates established and investigational HER2-directed therapies, with special attention to central nervous system involvement and ongoing clinical trials that may further redefine standards of care. Key clinical trials evaluating HER2-targeted therapies in NSCLC are summarized in **Tables 1**–**3**.

**Table 1:** Clinical activity of HER2-targeted tyrosine kinase inhibitors in HER2-altered non-small cell lung cancer.

Agent	Mechanism	Study	Population	ORR	mPFS	Safety
Lapatinib	Dual EGFR/HER2 (reversible)	Phase II	HER2-mutant	<5%	–	Rash, diarrhea
Pyrotinib	Dual EGFR/HER2 (irreversible)	Phase II/PYRAMID-1 (ongoing)	HER2-mutant	20%-50%	~6	Diarrhea, rash
Dacomatinib	Pan-HER (irreversible)	Phase II	HER2-mutant/amplified	12%	3	Diarrhea, rash
Neratinib +/- temsirolimus/ trastuzumab	Pan-HER (irreversible)	Phase II	HER2-mutant	8%	2.9-5.4	Diarrhea
Afatinib	Pan-HER (irreversible)	NICHE/Phase II	HER2-mutant	7%	3.7	Diarrhea, rash
Poziotinib	EGFR/HER2 exon 20 inhibitor	ZENITH-20 Phase II PINNACLE Phase III	HER2 exon20-mutant	28%-39%	5.5-5.6	Diarrhea, rash
Tarloxitinib	Hypoxia-activated pan-HER (IV)	RAIN-701 Phase II	HER2-mutant	22%	–	QTc prolongation, rash
Tucatinib	Selective HER2 (reversible)	SGNTUC-019 Phase II	HER2-altered	ongoing	–	Diarrhea
Zongertinib	Next-generation selective HER2 exon 20	BEAMION – LUNG Phase I/II	HER2-mutant	77%	–	GI, dermatologic
Sevabertinib	Selective HER2 (reversible)	SOHO-1 Phase I/II	HER2-mutant	64% (pretreated) 38% (post-ADC) %71 (naïve)	9.2 8.5 11	Rash, diarrhea

**Table 2:** Clinical efficacy and safety of HER2-directed ADCs in HER2-altered NSCLC.

Agent	Antibody/Payload/Linker	Study	Population	ORR	mPFS	mOS	Safety
Trastuzumab Emtansine (T-DM1)	Trastuzumab + DM1 (microtubule inhibitor), non-cleavable linker	Phase II	HER2-overexpressing/mutant NSCLC	6–20%	2-3	11-12	Mild hepatic AEs, thrombocytopenia
Trastuzumab Deruxtecan (T-DXd)	Trastuzumab + DXd (topoisomerase I inhibitor), cleavable linker	DESTINY	HER2-mutant NSCLC, post-TKI/chemo	49–56%	9-15	18-19	ILD 12–28%, neutropenia
Trastuzumab Rezetecan (SHR-A1811)	Trastuzumab + Topo I inhibitor, cleavable linker	HORİZON	Pretreated HER2-mutant NSCLC	73%	11.5	NE	Mild ILD (<5%)
Disitamab Vedotin (RC48)	Humanized anti-HER2 + MMAE (microtubule), mc-vc linker	RESOLOTIUN	HER2-altered NSCLC (± platinum ± bevacizumab)	45–71%	7.5	NE	Fatigue, neuropathy, no ILD

**Table 3:** Clinical outcomes of immunotherapy-based strategies in HER2-altered NSCLC.

Treatment	Study	Patient population	Line of therapy	ORR/PFS	Notes
ICI monotherapy (PD-1/PD-L1 inhibitors)	Retrospective cohorts (multiple studies) (89, 92)	HER2-mutant/HER2-altered NSCLC	Mostly ≥2L	ORR 7–12%; median PFS ~3–4 months	Inferior outcomes compared with unselected NSCLC
ICIs (mono or combination)	Systematic review & meta-analysis (96)	HER2-mutant NSCLC	Mixed	Pooled ORR ~20–25%; median PFS ~3–5 months	Lowest benefit observed with ICI monotherapy
Trastuzumab deruxtecan + durvalumab	DESTINY-Lung03 (NCT04686305) (98) Phase Ib	HER2-expressing NSCLC	Advanced	NR	Study ongoing; results not yet fully reported
Trastuzumab deruxtecan + pembrolizumab	DS8201a-U106 (99) Phase Ib	HER2-expressing or HER2-mutant NSCLC (ICI-naïve)	Advanced	ORR 54.5% (HER2-expressing); 66.7% (HER2-mutant); median PFS ~15 months	Preliminary results; early-phase, non-randomized
EGFR TKI + ICI	Historical EGFR TKI + ICI trials (100) Phase I-II	EGFR-mutant NSCLC	Advanced	NR	High pneumonitis rates led to early trial termination
TDXd+ pembrolizumab	DESTINY-Lung06 (NCT06899126) (103) Phase III	HER2-overexpressing NSCLC, PD-L1 <50%	1L	NR	Randomized trial; results pending

## 2 Molecular subtypes, activation mechanisms, and diagnostic standards

HER2 is activated in NSCLC through three distinct molecular mechanisms: gene mutation, gene amplification, and protein overexpression. Each of these mechanisms differs in terms of biological behaviour and therapeutic predictability.

### 2.1 HER2 mutations

HER2 mutations occur in approximately 2–4% of NSCLC cases, with slightly higher rates reported in Asian populations (~4%) than in Europe and North America (~2%) (6, 12). These mutations are more common in women, Asians, and never-smokers, closely mirroring the epidemiologic profile of EGFR-mutated NSCLC (13).

HER2 mutations are diverse, including single-nucleotide variants, insertions, deletions, and gene fusions, and can be localized to the extracellular, transmembrane, juxtamembrane, or kinase domains (14). In NSCLC, most clinically relevant mutations occur in the kinase domain as in-frame exon 20 insertions (15). Exon 20 insertions account for roughly 34-83% of HER2 mutations (16). Crucially, the spectrum of these exon 20 insertions is less heterogeneous than other oncogenic drivers; the A775_G776insYVMA variant—often termed the G776YVMA insertion—is the predominant alteration, itself responsible for nearly half of all HER2 mutations in NSCLC (17). This variant constitutively activates the receptor and its downstream signaling, promoting oncogenic transformation. Significantly, HER2 exon 20 insertions are less heterogeneous than other exon 20 alterations, as the vast majority involve a single recurrent event: a 12-base pair duplication of the Tyr-Val-Met-Ala (YVMA) motif at codon 775 (A775_G776insYVMA), which constitutes the predominant HER2 insertion mutation (18). In addition to exon 20 insertions, recurrent HER2 point mutations represent a clinically relevant subset of HER2-altered NSCLC. Among these, exon 8 S310F/Y mutations located in the extracellular domain are among the most frequently reported point mutations and constitute one of the most common HER2 aberrations following exon 20 insertions such as G776delinsYVMA. These alterations may display distinct biological behavior and therapeutic sensitivity compared with exon 20 insertions (19). Furthermore, HER2 mutations are usually mutually exclusive with mutations in other oncogenic drivers such as EGFR, KRAS, BRAF, and ALK rearrangements (6). On the other hand, some HER2 alterations (particularly amplification) act as an acquired mechanism of resistance to EGFR tyrosine kinase inhibitor (TKI) treatment (20).

Less commonly, mutations that activate the HER2 pathway have also been identified in the transmembrane and juxtamembrane domains. Examples include G660D, R678Q, E693K, and Q709L (21). About 8% of HER2 mutations are point mutations, such as L755S, D769H, V777L, and V777M (6).

### 2.2 HER2 amplifications

*De novo* ERBB2 amplification is detected in 2–5% of untreated NSCLC, whereas acquired amplification contributes to up to 10% of resistance events after TKI therapy (22). HER2 amplification demonstrates heterogeneous clinic-pathologic associations across studies. Some reports indicate enrichment among smokers, while others describe a distribution similar to HER2-mutant cases, predominantly found in female never-smokers (5, 17, 23). Although no universal definition exists, HER2 amplification is most commonly characterized by a HER2/CEP17 ratio ≥ 2.0 by fluorescence *in situ* hybridization (FISH). True amplification must be distinguished from copy number gain (CNG), which often reflects chromosome 17 polysomy rather than a driver alteration (24, 25).

### 2.3 HER2 overexpression

The incidence rate of HER2 overexpression is reported with a wide range of 7%-23% in NSCLC (26). HER2 overexpression refers to elevated HER2 protein levels on the tumor cell membrane, typically measured by immunohistochemistry (IHC) with gastric cancer scoring criteria (0, 1+, 2+, 3+) (27). HER2 overexpression is reported more frequently in males and smokers (23).

HER2 overexpression has been associated with poor prognosis (28). Nakamura et al. analyzed 2, 579 patients and found that HER2-positive NSCLC was associated with significantly lower survival rates, regardless of disease stage (29). Similarly, Liu et al. reviewed 14 studies involving 6, 135 patients and confirmed that HER2 overexpression is linked to worse prognosis (30). While the precise cause remains unclear, HER2 overexpression influences cell adhesion and invasive cancer growth by interacting with the cadherin-catenin complex (31).

**Figure 3:**
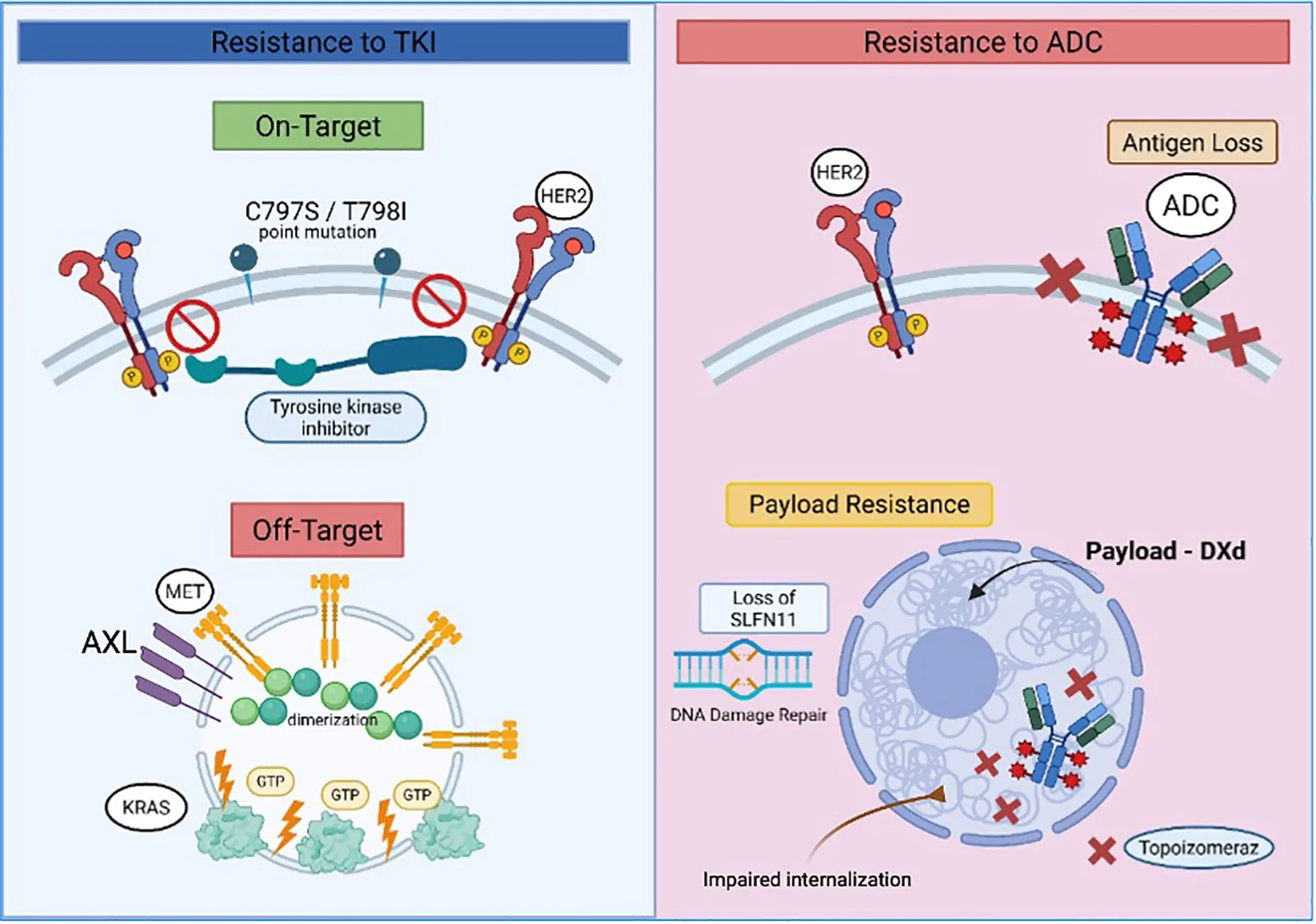
Resistance mechanisms against TKIs and ADCs.

HER2 mutation, amplification, and overexpression represent distinct molecular mechanisms, each resulting in different biological and clinical outcomes (**Figure 3**). While amplification/overexpression alone rarely predicts benefit from targeted agents, the presence of a HER2 exon 20 insertion is the primary predictive biomarker for response to HER2-directed therapies.

## 3 Molecular HER2 alterations testing strategies

Multiple methodologies are utilized to detect HER2 mutations, gene amplifications, and protein overexpression, with each approach offering specific strengths and limitations. HER2 status can be evaluated using tumor tissue samples, cytologic specimens, or circulating tumor DNA (ctDNA) to inform clinical decision-making (27).

### 3.1 HER2 mutation test

Sanger sequencing was historically used for HER2 mutation analysis and continues to serve as a validation tool for novel variants. However, its low sensitivity and limited utility in small biopsy samples have led to its replacement by more sensitive and comprehensive methods (32).

Amplification Refractory Mutation System-PCR (ARMS-PCR) offers high sensitivity, specificity, and operational simplicity; however, it cannot detect unknown mutations, and when multiple mutation sites need to be tested, DNA input requirements increase along with the risk of non-specific binding (27, 33).

Droplet digital PCR (ddPCR) offers accurate quantification of DNA mutations with high sensitivity, especially for detecting low-frequency variants. It is particularly effective for monitoring known mutations, although its benefits are less significant in samples with high DNA concentrations (28).

Next-generation sequencing (NGS) sequences millions of templates concurrently, requiring relatively little DNA while detecting exon 20 (YVMA and non-YVMA) insertions, missense mutations, copy number variations, and amplification with high sensitivity. An optimal NSCLC panel should capture all clinically relevant HER2 alterations with high reproducibility and rapid turnaround (17, 34).

### 3.2 HER2 amplification test

HER2 amplification in NSCLC is most often evaluated by fluorescence *in situ* hybridization (FISH) or NGS. FISH, the reference standard, defines amplification as a HER2/CEP17 ratio ≥ 2.0 or ≥ 6 copies per nucleus according to ASCO/CAP and IASLC criteria. NGS can simultaneously detect copy-number changes and co-occurring driver mutations but currently lacks standardized thresholds for defining amplification, so confirmation by FISH is recommended for ambiguous results. Chromogenic *in situ* hybridization (CISH) detects ERBB2 copy gain through chromogenic probe labeling visualized under a standard bright-field microscope (35). It offers comparable sensitivity to FISH but with easier interpretation in relation to tumor morphology and the advantage of long-term slide storage. Nevertheless, signal contrast is lower than FISH, and inter-observer variability can occur in borderline cases.

### 3.3 HER2 overexpression test

According to current NCCN and ASCO guidelines, HER2 overexpression in NSCLC should be assessed by IHC, adapting the gastric cancer scoring criteria due to the similar characteristics of tumor heterogeneity and staining patterns observed in lung cancer.

However, even with adapted scoring, HER2 amplification and overexpression show poor concordance in NSCLC, with a notably low agreement in the IHC 2+ subgroup and limited sensitivity of IHC for detecting amplification. Crucially, this low agreement in the IHC 2+ subgroup means FISH confirmation for these cases is not routinely recommended. Based on current evidence, HER2 expression in NSCLC is categorized as follows: 0/1+ is considered negative (though 1+ may be reconsidered as “HER2-low”); and 2+/3+ is considered positive (27).

#### 3.3.1 HER2”Low” in NSCLC

The significance of “HER2-low” disease in NSCLC is uncertain and different from its established role in breast cancer (36). Although HER2-low definition has gained exploratory interest, largely due to the success of ADCs in HER2-low breast cancer, its application in lung cancer is not yet clinically validated. In breast cancer, tumors with low HER2 expression (IHC 1+ or 2+ without gene amplification) however HER2 expression in NSCLC is highly variable, lack standardized scoring criteria, and shows poor concordance with gene amplification (25, 37). As noted previously, the ESMO consensus for the assessment of HER2 alterations in NSCLC has affirmed that an IHC 1+ score can be considered negative or low, while IHC 2+ and 3+ scores should be considered positive. However, current evidence suggests that HER2 protein overexpression alone does not reliably predict a significant response to HER2-directed therapies in NSCLC. ADC efficacy is primarily determined by the presence of HER2-activating mutations rather than protein expression levels (38).

## 4 Therapeutic advances

The current standard first-line treatment for advanced NSCLC with HER2 alterations remains platinum-based chemotherapy combined with immune checkpoint inhibitors (ICIs). However, despite multiple clinical trials, these regimens have not yet been replaced as the standard of care. Early studies evaluating HER2-targeted therapies yielded disappointing outcomes, but the emergence of antibody–drug conjugates (ADCs) has dramatically reshaped the therapeutic landscape.

HER2-directed therapy continues to represent a major focus of clinical research and drug development in NSCLC, given the gene’s role as a key oncogenic driver. Therapeutic strategies targeting HER2 can be broadly categorized into four main classes: monoclonal antibodies, tyrosine kinase inhibitors (TKIs), ADCs, and bispecific antibodies.

### 4.1 Monoclonal antibodies

HER2-targeted monoclonal antibodies (mAbs) bind to the extracellular domain of HER2, leading to receptor downregulation and inhibition of downstream signaling. By blocking dimerization, they suppress the PI3K/AKT and MAPK pathways, thereby reducing tumor cell proliferation and survival. In addition, these antibodies trigger antibody-dependent cellular cytotoxicity (ADCC), recruiting natural killer cells to attack HER2-expressing tumor cells. Binding to domain II of HER2 also prevents its heterodimerization with HER3 and HER1, further silencing growth-promoting signals (39). Despite the strong biological rationale, clinical trials of HER2-targeted monoclonal antibodies in NSCLC have shown limited benefit, in contrast to their proven efficacy in HER2-positive breast and gastric cancers.

Trastuzumab is a humanized monoclonal immunoglobulin G1 antibody derived from murine sequences, targets the HER2 extracellular domain and prevents receptor dimerization. Initial investigations HER2 targeted in NSCLC began in the early 2000s. Early studies of patients with HER2-overexpressing NSCLC treated with trastuzumab plus docetaxel, carboplatin/paclitaxel, or cisplatin/gemcitabine demonstrated variable and generally modest results (40, 41). A randomized phase II trial by Gatzemeier et al. evaluated gemcitabine–cisplatin with or without trastuzumab in patients with HER2-overexpressing advanced NSCL; trastuzumab failed to improve response or survival compared with chemotherapy alone (42).

Subsequent clinical studies to identify subgroups that might derive greater benefit focused on patients with HER2 amplification or mutation rather than protein overexpression. The HOT1303-B phase II trial evaluated trastuzumab monotherapy in a molecularly enriched cohort of patients with HER2-altered NSCLC (defined as IHC 2+/3+ overexpression and/or HER2 mutations) who had received at least two prior lines of systemic therapy. However, no objective responses were observed, either among patients with HER2 overexpression or those harboring HER2 mutations, reaffirming the limited therapeutic efficacy of HER2-directed monoclonal antibody monotherapy in NSCLC (43). The limited efficacy of first-generation anti-HER2 monoclonal antibodies is often attributed to comparatively lower HER2 expression in NSCLC and the structural difficulty in blocking the constitutive activation driven by Exon 20 insertion mutations.

Pertuzumab is a humanized monoclonal antibody that targets the extracellular domain II of HER2. It complements trastuzumab by inhibiting ligand-dependent HER2–HER3 dimerization and decreasing signaling through pathways such as PI3K/Akt (44). Later, dual HER2 inhibition with trastuzumab plus pertuzumab, with or without docetaxel, was explored in small cohorts of HER2-altered NSCLC, producing only modest efficacy (ORR 8–29%) (45, 46).

These findings underscored the need for alternative HER2-directed approaches, leading to the development of small-molecule TKIs and ADCs with enhanced potency and selectivity.

### 4.2 Tyrosine kinase inhibitors

HER2-targeted TKI therapy has evolved through several generations, reflecting a progressive shift from non-selective dual inhibitors to highly specific agents designed to optimize efficacy and tolerability.

#### 4.2.1 Early-generation and pan-HER inhibitors

Lapatinib, the first HER2-targeted tyrosine kinase inhibitor evaluated in NSCLC, is a reversible dual EGFR/HER2 inhibitor. In a phase II trial, it failed to demonstrate clinical activity in patients with HER2-mutant NSCLC, leading to discontinuation of its development for this indication (47). Subsequently, pan-HER inhibitors—including pyrotinib, dacomitinib, neratinib, afatinib, poziotinib, and tarloxotinib—were developed to achieve broader ERBB blockade, though most were constrained by modest response rates and substantial toxicity.

Pyrotinib, an irreversible dual EGFR/HER2 inhibitor, produced response rates of 20–50% and median PFS of approximately 6 months across phase II studies, with diarrhea and rash as the predominant toxicities (48, 49),. The ongoing phase III PYRAMID-1 trial (NCT04447118) comparing pyrotinib with docetaxel has completed enrollment. Dacomitinib, an irreversible pan-HER TKI, showed limited efficacy in a phase II trial (ORR 12%, mPFS 3 months, mOS 9 months) with frequent diarrhea and rash (50). Neratinib, as monotherapy or combined with temsirolimus or trastuzumab, produced low response rates (0–8%) and short PFS (2.9–5.4 months), with gastrointestinal adverse events being predominant (51–53). Afatinib, another irreversible inhibitor, yielded minimal benefit (ORR 7%, mPFS 3.7 months) in the NICHE and other phase II studies, again limited by diarrhea and rash (54–56). Poziotinib demonstrated higher activity in the ZENITH20 phase II trial (ORR 28–39%, mPFS 5.5–5.6 months) but was associated with substantial grade ≥3 rash and diarrhea, leading to frequent dose reductions and the early termination of its planned phase III PINNACLE trial due to tolerability concerns (57–59). In a phase II trial of tarloxotinib, an intravenously administered hypoxia-activated pan-HER inhibitor, preliminary results in HER2-mutant NSCLC showed modest activity with an ORR of 22% and DCR of 67%, though frequent QTc prolongation and rash limited its tolerability (60, 61). Collectively, these experiences underscored the narrow therapeutic index of earlier pan-HER TKIs, in which EGFR cross-inhibition often limited clinical feasibility.

#### 4.2.2 Next-generation selective HER2 inhibitors

The focus subsequently shifted toward selective HER2 inhibitors with greater molecular precision and reduced off-target toxicity. Tucatinib, a highly selective and reversible HER2 inhibitor, is currently being evaluated in the phase II SGNTUC-019 trial, a pan-tumor study investigating tucatinib-based therapeutic strategies across HER2-altered solid tumors, including dedicated cohorts of patients with non–small cell lung cancer (62).

More recently, zongertinib has emerged as one of the most promising agents in this class. Designed to selectively target HER2 exon 20 insertions while sparing wild-type EGFR, zongertinib has shown encouraging efficacy in early studies, achieving ORR > 40% and a favorable safety profile (63). Updated data from the Beamion LUNG-1 trial reported ORR 77% and DCR 96% among treatment-naïve HER2-mutant NSCLC patients, including those with brain metastases, with predominantly low-grade gastrointestinal and dermatologic events. These findings support its ongoing phase III evaluation (Beamion LUNG-2) against standard chemotherapy ± immunotherapy. In August 2025, the U.S. Food and Drug Administration (FDA) approved zongertinib based on the results of the phase 1/2 Beamion LUNG-1 study (NCT04886804), marking a major therapeutic advance for patients with previously limited treatment options (64).

In parallel, sevabertinib, a potent, reversible oral tyrosine kinase inhibitor with activity against both HER2 and EGFR, has demonstrated robust and durable efficacy in the SOHO-01 trial. Among 209 patients with HER2-mutant NSCLC, ORRs reached 64% in previously treated, 38% post-ADC, and 71% in treatment-naïve cohorts, with median durations of response of 9.2, 8.5, and 11 months, respectively. Toxicities were manageable (mostly grade 1–2 diarrhea and rash), and importantly, no interstitial lung disease was observed (65). In November 2025, FDA granted accelerated approval to sevabertinib for patients with previously treated, locally advanced or metastatic non-squamous NSCLC harboring HER2 (ERBB2) tyrosine kinase domain–activating mutations (66).

From a mechanistic perspective, zongertinib is designed as a covalent, irreversible HER2-selective tyrosine kinase inhibitor, whereas sevabertinib is a reversible dual EGFR/HER2 TKI. These pharmacologic differences may translate into distinct efficacy and safety profiles, particularly with respect to on-target EGFR-related toxicities.

Collectively, these results significant a pivotal shift from broad ERBB blockade toward precise HER2 inhibition. Next-generation TKIs such as zongertinib and sevabertinib provide substantial antitumor activity with improved tolerebility across multiple treatment settings, representing a meaningful step forward in the therapeutic landscape of HER2-mutant NSCLC. Nevertheless, response durability remains variable, underscoring the continued need for complementary therapeutic strategies, including both antibody–drug conjugates and next-generation kinase inhibitors, to enhance long-term disease control and address mechanisms of resistance.

### 4.3 Antibody-drug conjugates

HER2 alterations in NSCLC encompass a heterogeneous spectrum, including exon 20 insertions, non–exon 20 point mutations, gene amplification, and protein overexpression. However, the most robust clinical evidence supporting antibody–drug conjugates in NSCLC has been generated predominantly in HER2-mutant disease, particularly driven by exon 20 insertions, while data for other mutation subtypes as well as for HER2 amplification and overexpression remain more limited and heterogeneous across studies.

**Figure 4:**
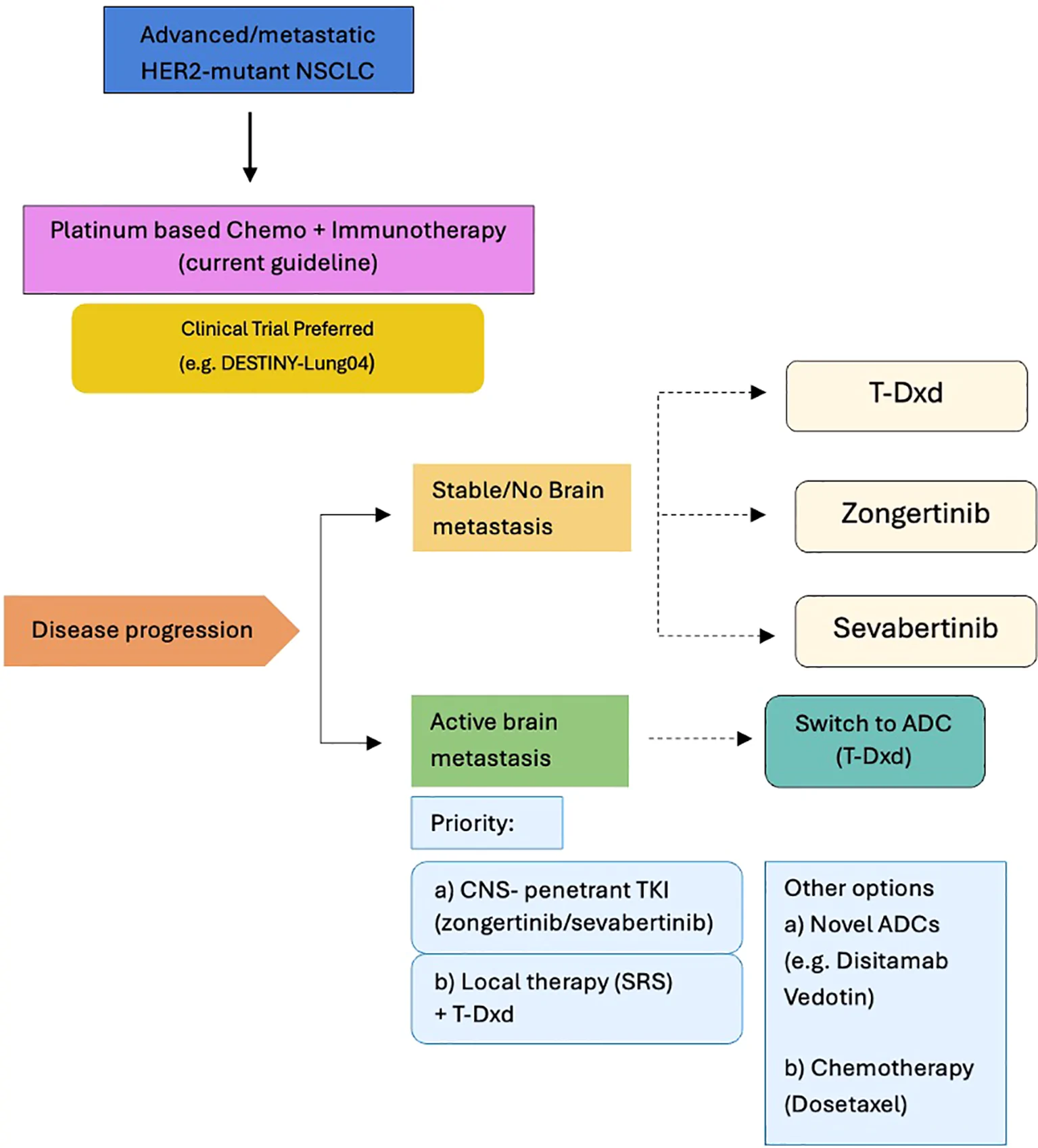
Current recommended treatment algorithm for HER2-Mutant NSCLC (2025).

Following the modest and short-lived responses observed with HER2-directed TKIs, ADCs have emerged as a transformative therapeutic class in HER2-mutant NSCLC. By coupling the target specificity of monoclonal antibodies with the potent cytotoxicity of chemotherapy, ADCs achieve precise intratumoral delivery while limiting systemic exposure. Each ADC consists of three key components: a monoclonal antibody directed against a tumor-associated antigen, a cleavable or non-cleavable linker, and a cytotoxic payload (**Figure 4**). In HER2-altered NSCLC, this design enables selective elimination of tumor cells expressing HER2, independent of membrane overexpression level, while retaining immune-effector activity such as antibody-dependent cellular cytotoxicity (ADCC) and generating the crucial “bystander effect” due to the membrane-permeable payload. Collectively, these agents have reshaped the treatment landscape, offering clinically meaningful activity even in heavily pre-treated patients and establishing HER2-mutant NSCLC as a distinct, targetable molecular subtype (67, 68).

Key structural determinants of antibody–drug conjugates include the drug-to-antibody ratio (DAR) and the conjugation strategy used to link the cytotoxic payload to the monoclonal antibody. Higher DAR may enhance payload delivery but can adversely affect pharmacokinetics and tolerability. In addition, site-specific conjugation results in more homogeneous ADC species, whereas non-specific (stochastic) conjugation generates heterogeneous constructs, potentially influencing linker stability, off-target payload release, and the balance between efficacy and toxicity (69, 70).

#### 4.3.1 Trastuzumab emtansine (T-DM1)

Ado-trastuzumab emtansine (T-DM1) is a HER2-targeted antibody-drug conjugate that combines trastuzumab with the microtubule inhibitor emtansine. In a multicenter phase II study, T-DM1 monotherapy demonstrated limited efficacy in HER2-positive NSCLC. Among 15 patients (IHC 3+, IHC 2+/FISH-positive, or exon 20 mutant), only one achieved a partial response (ORR 6.7%). Median progression-free survival was 2.0 months, and median overall survival was 10.9 months. The treatment was generally well tolerated, but the trial was terminated early due to limited efficacy. These findings suggested that HER2 overexpression or amplification alone may not be sufficient to predict benefit from HER2-directed ADCs in NSCLC (71). This trial was conducted primarily in a Japanese patient population. In a subsequent phase II study by Peters et al. with a larger multicenter cohort, T-DM1 achieved an ORR of 20% and median PFS and OS of 2.6 and 12.2 months, respectively, in patients with HER2 IHC 3+ NSCLC, confirming its limited efficacy in this setting (72).

The clinical failure of T-DM1 in NSCLC is primarily attributed to insufficient HER2 antigen expression and intrinsic pharmacological design constraints. Low and heterogeneous HER2 expression in lung tumors impedes effective target engagement, while the structural characteristics of T-DM1 further limit its cytotoxic potential. T-DM1 links trastuzumab to the maytansinoid payload DM1 via a non-cleavable thioether linker, necessitating complete lysosomal degradation for payload release. In HER2-mutant NSCLC, inefficient internalization and trafficking hinder this process, resulting in inadequate intracellular drug delivery. Additionally, the generated metabolite is membrane-impermeable, which prevents diffusion to neighboring tumor cells and abolishes the bystander killing effect (72). These pharmacological limitations have been overcome by next-generation ADCs, which utilize cleavable linkers and membrane-permeable payloads to facilitate broader drug release and effective bystander killing, even in heterogeneous tumors (73).

#### 4.3.2 Trastuzumab deruxtecan

Trastuzumab Deruxtecan (T-DXd) is structurally composed of the humanized anti-HER2 IgG1 monoclonal antibody, Trastuzumab, conjugated to a potent topoisomerase I inhibitor payload, Deruxtecan (DXd), via a tetra-peptide-based cleavable linker (74). This phase I dose-expansion study, which excluded breast and gastric cancers due to established HER2-targeted therapy benefits, showed promising efficacy in the NSCLC cohort compared to other solid tumor types investigated (74). This finding delivered the first clinical proof of concept for T-DXd in HER2-mutant NSCLC, demonstrating its potential as an actionable targeted therapy.

The pivotal DESTINY-Lung01 phase II trial demonstrated that T-DXd achieved an objective response rate of 54.9% and a median progression-free survival of 8.2 months and a median overall survival 17.8 months at the 6.4 mg/kg dose in previously treated patients with HER2-mutant NSCLC (75). Although the incidence of grade 3 or higher adverse events was 46 percent, with neutropenia being the most common at 19 percent. A significant number of ILD cases were observed, accounting for 26%. One in four patients discontinued treatment, with most cases linked to pneumonitis or ILD (75). Due to the relatively high incidence of interstitial lung disease observed in the initial DESTINY-Lung01 trial, a follow-up study was designed to identify a safer dose while maintaining efficacy.

T-DXd incorporates several structural innovations that enhance its therapeutic efficacy. It has a high drug-to-antibody ratio (DAR ≈ 8:1), delivering more cytotoxic payload per antibody while maintaining stability and pharmacokinetics (73, 76). The cleavable tetrapeptide linker is selectively degraded by lysosomal proteases such as cathepsins, ensuring efficient intracellular payload release (73). Its membrane-permeable topoisomerase I inhibitor (DXd) diffuses into neighboring tumor cells, producing a strong bystander effect even in tumors with heterogeneous or low HER2 expression (73, 74). Collectively, these pharmacologic features translate into deeper and more durable responses in HER2-expressing solid tumors, including HER2-mutant NSCLC.

The DESTINY-Lung02 (DL-02) trial was a randomized Phase 2 study specifically designed to assess the efficacy and safety of T-DXd at two lower doses, 5.4 mg/kg and 6.4 mg/kg, in patients with previously treated HER2-mutant NSCLC (77). Among 152 enrolled patients, confirmed objective response rates were 49% and 56%, with median durations of response of 16.8 months and not estimable, and median progression-free survival of 9.9 and 15.4 months in the 5.4 mg/kg and 6.4 mg/kg cohorts, respectively. Median overall survival reached 19.5 months with the lower dose. Interstitial lung disease occurred in 12.9% of patients receiving 5.4 mg/kg and 28% at 6.4 mg/kg, confirming a more favorable safety profile at the recommended 5.4 mg/kg dose (77). Consequently, the 5.4 mg/kg dose has been established as the recommended regimen for T-DXd in HER2-mutant NSCLC leading to its subsequent accelerated approval by the U.S. FDA on August 11, 2022, based on the compelling response data and favorable risk-benefit balance demonstrated by this dose (11). The approval was supported by interim data from DESTINY-Lung02, which confirmed durable efficacy and improved tolerability at the 5.4 mg/kg dose, marking the first FDA-approved therapy specifically for HER2-mutant NSCLC (11).

Next came DESTINY-Lung04, an ongoing open-label phase III trial for patients with HER2-altered NSCLC receiving first-line treatment (78). Beyond its robust efficacy in the HER2-mutant population, T-DXd has fundamentally challenged the traditional definition of HER2 positivity in NSCLC. The pronounced bystander effect, enabled by its cleavable linker and membrane-permeable payload, facilitates activity even in tumors classified as HER2-Low (IHC 1+ or IHC 2+/FISH-negative). This expands the actionable patient population and underscores a key distinction between T-DXd and prior HER2-directed agents, necessitating a reconsideration of standard HER2 testing algorithms solely focused on mutation or IHC 3+ status (74, 79),.

#### 4.3.3 Novel ADCs

Trastuzumab rezetecan (SHR-A1811) is a novel ADC composed of a monoclonal antibody, a cleavable tetrapeptide linker, and a DNA topoisomerase I inhibitor. In the phase II HORIZON-Lung study conducted in China, 94 pretreated patients with HER2-mutant NSCLC were enrolled. The study reported an ORR of 73% and a median progression-free survival (mPFS) of 11.5 months. OS data were not yet mature. The safety profile was manageable, with low rates of interstitial lung disease (80).

Disitamab vedotin (DV or RC48) is composed of three key components: a humanised IgG1 monoclonal antibody targeting HER2, the microtubule-disrupting agent MMAE (monomethyl auristatin E), and a protease-cleavable mc-vc (maleimidocaproyl-valinecitrulline) linker that covalently binds MMAE to the antibody. It was well tolerated and demonstrated efficacy in several HER2-positive cancers, including breast, gastric, and urothelial carcinomas (81–83). A recent multicenter retrospective study in China looked at 22 patients with HER2-altered advanced NSCLC who received disitamab vedotin, mostly as part of combination treatments. The overall response rate was 45.5%, the disease control rate was 90.9%, and the median progression-free survival was 7.5 months. The best results were seen with RC48 plus  nbsp;platinum, with or without bevacizumab, showing a 71.4% response rate. The treatment was generally well-tolerated, with no cases of grade 3 or higher ILD and only one grade 3 adverse event (4.5%) (84). Phase II clinical trials of RC48 in HER2-altered NSCLC are ongoing to further validate these findings (NCT0674986, NCT05847764). In the RESOLUTION phase II trial (NCT06749860), the first-stage interim analysis of disitamab vedotin combined with tislelizumab and bevacizumab in treatment-naïve HER2-altered NSCLC (n = 9) demonstrated an ORR of 33.3%, median duration of response of 5.1 months, and manageable safety profile (no ILD, grade 3 AEs = 22%). These data met the futility threshold and supported continuation to the next stage of the trial (85).

Trastuzumab rezetecan and disitamab vedotin have been primarily investigated in China, and the majority of available clinical evidence derives from Asia–Pacific cohorts. These data, although regionally limited, highlight the expanding global interest in HER2-directed ADCs beyond Western populations.

Several novel HER2-directed ADCs, including BL-M17D1(NCT06114511), TQB2102(NCT06496490), and MRG002 (NCT05141786), are currently being explored in early-phase trials, along with multi-tumor candidates such as DB -1303(NCT05150691), XMT-2056(NCT05514717), and GQ1001(NCT04450732).

### 4.4 Bispecific antibodies

Bispecific antibodies (bsAbs) are engineered immunoglobulins capable of binding two different epitopes or antigens simultaneously, enabling dual-pathway inhibition or immune-cell engagement. By combining target selectivity and immune activation in a single molecule, bsAbs can effectively block cooperative receptor signaling and overcome resistance observed with conventional monoclonal antibodies (86).

Zanidatamab (ZW25) is a biparatopic bispecific antibody that binds two distinct HER2 epitopes (domains II and IV), promoting receptor clustering and internalization. This dual binding enhances HER2 signal blockade and immune effector functions such as ADCC, leading to durable antitumor activity. In early-phase studies, zanidatamab achieved response rates around 30–40% across HER2-positive solid tumors, including NSCLC, with a manageable safety profile (87).

Although not directly targeting HER2-mutant disease, the HER2×HER3 bispecific antibody Zenocutuzumab (MCLA-128) has demonstrated efficacy in NRG1 fusion–positive tumors by inhibiting HER3 activation and HER2–HER3 dimerization, highlighting the broader relevance of HER2-related signaling in oncogenic networks (88).

### 4.5 Immunotherapy

Immune checkpoint inhibitors (ICIs) have reshaped the therapeutic landscape of NSCLC, yet their efficacy in oncogene-driven tumors remains limited (89, 90). Emerging evidence suggests similarly modest activity in HER2-mutant disease (91). HER2-mutant NSCLC which typically arises in younger, never-smokers and exhibits low tumor mutational burden (TMB) and low PD-L1 expression, is increasingly characterized as an “immune cold” subtype (19, 92). These features correlate with poor responsiveness to ICIs.

#### 4.5.1 Limited activity of ICI monotherapy

Across retrospective and small prospective cohorts, HER2 altered NSCLC demonstrates modest sensitivity to mono-immunotherapy. ORRs range from 7–12% and median PFS remains 3–4 months consistent with pooled estimates from a recent metanalysis with ICI monotherapy. These results are markedly inferior to those observed in unselected NSCLC populations and comparable to or only marginally better than chemotherapy in this molecular subgroup (91, 93). In addition several analyses showed that ICI responses correlate with PD-L1 expression and TMB in HER2-mutant group, whereas no such associations were found in HER2 amplified group (91). However, all available evidence comes from retrospective studies, each with significant limitations, including small number of patients, heterogenous baseline characteristics such as smoking status, PD-L1 expression, HER2 alteration. Many immunotherapy trials excluded patients with actionable driver mutations, further limited evidence base. Consequently, the current evidence of ICI efficacy of HER2 altered NSCLC is based limited studies and highlightining the need robust prospective data.

#### 4.5.2 Tumor microenvironment and immune resistance

Emerging evidence shows that HER2 alterations shape the tumor microenvironment (TME) and effect antitumor immune responses. In HER2 positive breast cancer models, activated HER2 signaling modulates cytokine networks, alters antigen-presentation pathways, and regulates the density and functional activity of tumor-infiltrating immune cells. These findings support a direct immunomodulatory role for HER2 (94). However, a pan-cancer analysis by Wang et al. revealed that the HER2mutation is associated with features promoting anti-tumor immunity, such as elevated TMB and CD8+ T-cell infiltration, resulting in improved OS for patients treated with ICIs, although this efficacy exhibits heteorgenity across different tumor types (95). In NSCLC, genomic and transcriptomic profiling of HER2-mutated tumors has identified distinct immune signatures, such as lower tumor purity, increased stromal content, reduced cytotoxic T-cell infiltration, and differences in immune-related pathway expression compared to HER2-wild type tumors (96). Additionally, many HER2-driven NSCLCs arise in never- or light- smokers and display relatively low TMB, implying a lower neoantigen load than smoking associated tumors and thereby reducing immune visibility and ICI responses (96).

#### 4.5.3 Immunotherapy combination strategies: ADC-based approaches

A systematic review and meta-analysis evaluating ICI combination-based regimens in HER-2 mutated NSCLC demonstrated modest activity overall with, pooled objective response rates in the 20-25% and median PFS 3–5 months across included studies. Notably ICI monotherapy showed the lowest benefit, particularly in previously treated patients, highlighting the limited effectiveness of single agent immunotherapy in this molecular subgroup (97). Given the limited efficacy of ICIs as monotherapy, current approaches in HER2 altered NSCLC emphasize combination strategies or sequencing in HER2-targeted agents rather than relying on single agent immunotherapy. One promising avenue is combining immunotherapy with HER2-targeted drugs. Preclinical studies suggest ADCs like T-DXd can induce immunogenic cell death and potentiate an anti-tumor immune response by releasing tumor antigens (98, 99),. This provides a strong biological rationale for combining ADCs with immune-checkpoint blockade. Early clinical trials support this strategy The phase Ib DESTINY-Lung03 study, currently investigating the combinationof T-DXd plus durvalumab in HER2-expressing NSCLC (100). Another ongoing trial DS8201a-U106 investigates T-DXd + pembrolizumab in HER2-altered NSCLC. The interim analysis presented a poster, demonstrated promising activiy for the combination of TDxd and pembrolizumab in ICI naïve HER2-expressing or HER2-mutant NSCLC with ORRs of 54.5% and 66.7%, respectively, and durable responses Importantly, in ICI-naïve patients, the combination achieved a median PFS around 15 months, suggesting a potential additive therapeutic effect; however these results are preliminary and require confirmation in larger, peer-reviewed studies (101).

#### 4.5.4 TKI–ICI combinations

Combining TKIs with immunotherapy is approached cautiously due to toxicity observed in prior EGFR TKI + ICI combinations, which resulted in high rates of pneumonitis and led to early termination several trials (102). Current reports only describe using TKIs or ICIs sequential in practice. Consequently, rigorously designed prospective trials are required to assess the feasibility of HER2 TKI and ICI combination regimens.

#### 4.5.5 Emerging immunotherapeutic strategies beyond ICIs

Researchers are investigating novel immunotherapeutic approaches for HER2-driven cancers, such as vaccines targeting HER2 or adoptive T-cell therapies (CAR-T or TCR-engineered cells) directed at HER2 (103–105).

Preclinical HER2-targeted CAR-T cell studies in HER2-positive NSCLC have leveraged co-expressed tumor-associated proteins or chemokine receptor pathways to enhance tumor trafficking and antitumor activity, supporting the translational potential of multi-signal CAR-T designs in this molecular subset. In this context, Hu et al. reported a preclinical study in HER2-positive NSCLC demonstrating that HER2-targeted CAR T cells co-expressing the chemokine receptors CXCR5 and CCR6 showed enhanced antitumor activity, persistence, and tumor infiltrations (104). These findings suggest that chemokine-guided CAR T-cell strategies may represent an investigational immunotherapeutic approach in this molecular subgroup.

#### 4.5.6 Clinical implications and future perspectives

These approaches are still in early development for lung cancer and, while promising, their clinical relevance remains unproven. For now, in the absence of a clear benefit, immunotherapy is often de-prioritized in HER2-mutant NSCLC unless used in combination or if targeted options are exhausted. The immune resistance phenotype of HER2-mutant NSCLC underscores the need for multimodal treatment strategies. Targeted therapy should address the oncogenic driver, and immunotherapy can be integrated through its combination or sequencing to overcome tumor’s immune-evasive mechanisms. The ongoing phase III DESTINY-Lung06 trial (NCT06899126) is comparing T-DXd plus pembrolizumab versus pembrolizumab with platinum-based chemotherapy in HER2-overexpressing, PD-L1 < 50% NSCLC (106). Beyond HER2-directed strategies, numerous ADC-ICI combination trials are currently underway across multiple tumor types including breast, bladder, gastrointestinal and lung reflecting broad clinical interest in defining the therapeutic potential of these regimens (107). Current evidence indicates that single-agent immunotherapy provides limited benefit in HER2-mutant NSCLC, and its therapeutic role will need to be refined as results from ongoing combination trials emerge.

## 6 Molecular resistance mechanisms to HER2-targeted therapies

As HER2-targeted agents become widely used, acquired resistance is an emerging challenge. Experience from other oncogene-driven cancers (e.g. EGFR-mutant NSCLC) shows that tumors can evade targeted therapies via diverse mechanisms. In HER2-positive NSCLC, both TKI resistance and ADC resistance mechanisms have been identified or postulated.

### 6.1 Resistance to TKIs

#### 6.1.1 On-target HER2 mutations

Secondary mutations in the HER2 kinase domain can impair TKI binding through steric hindrance or increased ATP affinity. Although these mutations are well documented in breast cancer (e.g., the T798I “gatekeeper” mutation, analogous to EGFR T790M) (108), clinical evidence in HER2-mutant NSCLC limited. Other variants including L755S or T862A may also reduce effectiveness of selective HER2 inhibitors (109).

#### 6.1.2 Activation of bypass pathways

Tumors may reduce dependence on HER2 signaling through activation of alternative survival pathways. In HER2-positive breast cancer and preclinical HER2-driven models, reported bypass mechanisms include MET amplification, activation of the RAS–MAPK and PI3K–AKT pathways, and enhanced signaling through other ERBB family members such as EGFR or HER3 (110). While these mechanisms are well characterized in other HER2-driven malignancies, their clinical relevance in HER2-mutant NSCLC remains incompletely defined. In addition, AXL upregulation has been proposed as a bypass mechanism associated with acquired resistance and a more mesenchymal phenotype, highlighting its potential relevance as a mediator of treatment resistance (111, 112).

### 6.2 Resistance to ADCs

#### 6.2.1 HER2 target loss or modification

Prolonged exposure to HER2-directed antibody–drug conjugates may select for tumor cell populations with reduced HER2 expression or target modification, leading to impaired ADC binding and payload delivery. Reported mechanisms include antigen downregulation, HER2 extracellular domain shedding, and epitope-altering changes that reduce antibody binding, predominantly described in HER2-positive breast cancer models (113). While these processes have not been systematically demonstrated in HER2-mutant NSCLC, their potential relevance cannot be excluded given the dependence of ADC efficacy on adequate HER2 surface expression.

#### 6.2.2 Impaired internalization or trafficking

The efficacy of antibody–drug conjugates depends on efficient internalization of the HER2–ADC complex and appropriate intracellular trafficking to enable payload release. In HER2-positive breast cancer and preclinical models, resistance has been associated with altered endocytic pathways or aberrant intracellular sequestration, resulting in reduced payload delivery and diminished cytotoxic activity (114). Although similar mechanisms may occur in NSCLC, clinical evidence remains limited.

#### 6.2.3 Drug efflux and metabolism

Upregulation of ATP-binding cassette (ABC) transporters, such as P-glycoprotein, can reduce intracellular concentrations of ADC-derived cytotoxic payloads through active drug efflux. This mechanism represents a recognized form of general chemoresistance and has been demonstrated in preclinical studies of various ADC payloads, where increased efflux pump expression is associated with reduced payload activity (115).

#### 6.2.4 Payload insensitivity

Resistance to antibody–drug conjugates may also arise from reduced tumor cell sensitivity to the cytotoxic payload itself. Trastuzumab deruxtecan delivers a topoisomerase I inhibitor, and tumor cells with enhanced DNA damage response or anti-apoptotic signaling may tolerate payload-induced DNA damage. Loss of SLFN11, a key determinant of sensitivity to DNA-damaging agents, has emerged as a biomarker associated with resistance to topoisomerase I–based therapies, including DXd-containing ADCs (116, 117). Additional payload-specific mechanisms, such as alterations in topoisomerase I, have been proposed but remain less well characterized.

### 6.3 Lineage plasticity

#### 6.3.1 Phenotypic transformation

Lineage plasticity is well documented in EGFR-mutant NSCLC, where adenocarcinomas can transform into small cell lung cancer under therapeutic pressure (118). In contrast, such phenotypic transformation appears to be rare and less well characterized in HER2-mutant NSCLC. Beyond small-cell transformation, squamous cell transformation has also been reported as a manifestation of lineage plasticity in oncogene-driven NSCLC, including cases with HER2 alterations, primarily in case reports and small series, and may complicate subsequent treatment selection (119, 120).

### 6.4 Microenvironment and cell survival pathways

Microenvironment and cell survival pathways: Tumor cells may activate cell-intrinsic survival and stress-response pathways following exposure to antibody–drug conjugates, leading to reduced sensitivity to cytotoxic payloads. Preclinical studies have linked altered apoptotic thresholds and activation of stress-response signaling to diminished ADC activity. In addition, components of the tumor microenvironment, including stromal and immune-derived signals, may modulate therapeutic responses by promoting tumor cell survival after cytotoxic injury. However, direct evidence supporting these mechanisms in resistance to HER2-directed ADCs in NSCLC remains limited (121).

### 6.5 HER2 alterations as an acquired resistance mechanism to other therapies

Beyond its role as a primary oncogenic driver, HER2 has also been identified as a mediator of therapeutic escape in other molecular subsets of NSCLC. Specifically, acquired HER2 amplification has been reported as a bypass resistance mechanism in approximately 2–10% of patients with EGFR-mutated NSCLC following treatment with third-generation EGFR TKIs such as Osimertinib(kaynak). In these settings, HER2 signaling can reactivate downstream PI3K–AKT and MAPK pathways despite continued EGFR inhibition. Emerging strategies to overcome this resistance include dual-targeted approaches, such as combining EGFR inhibition with HER2-directed antibody–drug conjugates (e.g., trastuzumab deruxtecan) or selective HER2 TKIs, which are currently being evaluated in early-phase clinical trials. While this paradigm is well established in EGFR-mutant disease, its relevance to HER2-mutant NSCLC remains to be defined.

Clinically, investigation of resistance to HER2-targeted therapies relies on repeat tumor biopsy or circulating tumor DNA (ctDNA) analysis at the time of disease progression, which may reveal emerging molecular alterations. To date, clinical evidence defining resistance mechanisms in HER2-mutant NSCLC remains limited, with much of the current understanding extrapolated from other HER2-driven malignancies and preclinical models. These observations highlight the biological heterogeneity of resistance and the need for systematic molecular characterization at progression. Given this complexity, future strategies are likely to emphasize rational treatment sequencing and the development of next-generation agents, rather than established combination approaches. A schematic overview of proposed resistance mechanisms across HER2-targeted therapies is provided in **Figure 3**.

## 7 Treatment sequencing and clinical management algorithm

The management of HER2-altered NSCLC is undergoing a paradigm shift, transitioning from a conventional “one-size-fits-all” approach toward a stage-specific, molecularly informed treatment model. Optimizing the sequence and integration of therapies is increasingly important to maximize long-term disease control while mitigating treatment-related toxicities.

In early-stage and locally advanced disease (stages I–III), ongoing research efforts are exploring the potential role of targeted and biomarker-driven strategies in the perioperative setting, although prospective HER2-specific data remain limited. In contrast, management of advanced and metastatic disease (stage IV) relies on the strategic sequencing of ADCs and next-generation TKIs.

This evolving landscape underscores the need for a nuanced, individualized treatment framework that integrates emerging clinical evidence with existing guideline recommendations. The following sections outline such an evidence-based approach, with treatment considerations for metastatic disease summarized in **Figure 4**.

### 7.1 Early-stage and locally advanced NSCLC (stage I–III)

At present, there are no approved adjuvant or neoadjuvant HER2-targeted therapies for early-stage disease. Management of resectable HER2-mutant NSCLC remains centered on definitive surgery followed by platinum-based chemotherapy, in accordance with current guideline recommendations. Radiotherapy is primarily used in medically inoperable or locally advanced disease and is not part of routine management in resectable early-stage NSCLC (122, 123).

#### 7.1.1 Immunotherapy considerations and sequencing risks

While perioperative immunotherapy has become a standard component of care for unselected early-stage NSCLC, its role in the HER2-mutant subset remains uncertain. Similar to EGFR- and ALK-driven disease, HER2-mutant tumors frequently exhibit an “immune-cold” phenotype, characterized by lower PD-L1 expression and tumor mutational burden, which has been associated with a more modest benefit from immunotherapy (91, 93).

In addition, treatment sequencing–related safety concerns merit consideration. Data from oncogene-driven NSCLC populations indicate that prior exposure to immune checkpoint inhibitors may increase the risk of severe interstitial lung disease or pneumonitis when patients subsequently receive targeted therapies, including HER2-directed ADCs or TKIs.

Accordingly, rather than routine application, the use of perioperative ICIs in HER2-mutant NSCLC should be individualized, ideally guided by multidisciplinary discussion and patient-specific risk assessment, with clinical trial participation encouraged whenever feasible.

#### 7.1.2 Emerging perioperative clinical trials

Several ongoing studies are evaluating whether molecularly targeted strategies can improve outcomes in the curative-intent setting. The phase III Beamion LUNG-03 trial (NCT07195695) is assessing adjuvant zongertinib compared with standard therapy in patients with completely resected stage II–IIIB HER2-mutant NSCLC (124). In parallel, NCT06734182 is a phase II neoadjuvant study investigating the combination of the ADC disitamab vedotin with chemotherapy in resectable HER2-mutant disease (125). Results from these trials will be critical in defining the future role of targeted and immunotherapy-based approaches in the perioperative management of HER2-mutant NSCLC.

### 7.2 Advanced and metastatic disease HER2-mutant NSCLC

#### 7.2.1 First-line therapy for advanced HER2-mutant NSCLC

Patients with advanced HER2-mutant NSCLC are generally treated with first-line regimens used for nonsquamous NSCLC, most commonly platinum-based chemotherapy combined with immunotherapy. In the absence of approved first-line HER2-targeted therapies, this remains the guideline-recommended first-line approach (84, 123). The ongoing phase III DESTINY-Lung04 trial is evaluating trastuzumab deruxtecan as first-line therapy compared with standard platinum-based chemo-immunotherapy in patients with advanced HER2-mutant NSCLC (78).

On the other hand, a growing clinical debate concerns the integration of ICIs in the first-line treatment of HER2-mutant NSCLC. In clinical practice, some clinicians may choose to de-emphasize ICIs or consider platinum-based chemotherapy alone in selected patients, drawing parallels to EGFR- and ALK-driven NSCLC, where single-agent ICI efficacy has been consistently modest (91, 94). This so-called “immune-cold” phenotype, characterized by lower tumor mutational burden and variable PD-L1 expression, has been associated with suboptimal responses to immunotherapy in oncogene-driven lung cancers (89, 93).

In addition to efficacy considerations, treatment sequencing–related safety concerns have emerged as an important factor. Data from EGFR TKI–treated populations indicate that prior ICI exposure is associated with an increased risk of severe pneumonitis when transitioning to targeted therapies (99). This issue may be particularly relevant in HER2-mutant NSCLC, given that both HER2-directed ADCs (such as trastuzumab deruxtecan) and certain TKIs carry an inherent risk of interstitial lung disease. Accordingly, in selected clinical scenarios, deferring ICIs may be considered to mitigate overlapping pulmonary toxicities and to preserve therapeutic flexibility for subsequent HER2-directed treatments.

#### 7.2.2 Second-line therapy for advanced HER2-mutant NSCLC

Following disease progression after platinum-based therapy, HER2-targeted therapies play a central role in managing patients with advanced HER2-mutant NSCLC (123). Current clinical practice guidelines, including recent NCCN updates, recognize trastuzumab deruxtecan, zongertinib, and sevabertinib as reasonable second-line options, without establishing a clear therapeutic hierarchy (123).

The absence of a preferred sequence reflects the lack of head-to-head randomized comparisons and the heterogeneity of study populations across available trials. Trastuzumab deruxtecan has demonstrated high response rates and durable disease control in HER2-mutant NSCLC, primarily driven by cohorts enriched for exon 20 insertions, albeit with a distinct toxicity profile, including the risk of interstitial lung disease. In contrast, selective HER2 TKIs such as zongertinib and sevabertinib offer an oral treatment option with differing pharmacologic properties and emerging evidence of central nervous system activity, which may be clinically relevant in selected patients. Cross-trial comparisons should be interpreted with caution due to differences in eligibility criteria, prior treatments, and biomarker definitions. Rather than favoring a single agent, treatment selection in the second-line setting should be individualized, taking into account disease burden and tempo, presence of brain metastases, comorbidities, prior toxicities, and patient preference. As additional data mature and practice guidelines continue to evolve, the optimal sequencing of ADCs and TKIs in HER2-mutant NSCLC remains an active area of investigation.

#### 7.2.3 Subsequent lines therapy for advanced HER2-mutant NSCLC

After utilizing both an ADC and a selective TKI, treatment options become more limited, but several pathways remain. Participation in clinical trials is strongly encouraged at this stage to access novel agents. Other investigational antibody–drug conjugates, including disitamab vedotin (RC48), are currently undergoing clinical evaluation. In a retrospective study from China with a limited sample size, RC48 achieved an objective response rate of 57.1% in patients who had received two prior lines of therapy. In contrast, the response rate was 14.3% among patients who had undergone three or more prior lines of treatment (126). In patients who have not previously received a HER2-directed therapy and for whom other HER2-targeted agents are unavailable or contraindicated, T-DM1 may be considered in selected circumstances; however, supporting evidence in this setting remains limited, and robust prospective data following prior HER2-directed therapy are lacking (72). In later lines, when HER2-directed options have been exhausted or are not feasible, management generally follows standard NSCLC approaches. Single-agent chemotherapy, such as docetaxel, with or without ramucirumab, may be considered according to clinical practice guidelines. Immunotherapy may be used in patients who have not previously received immune checkpoint inhibitors, although expected benefit is generally modest in oncogene-driven tumors. Supportive care measures and localized therapies for symptom control should be integrated as appropriate, and participation in clinical trials remains strongly encouraged whenever feasible.

#### 7.2.4 Guideline alignment for advanced HER2 mutant NSCLC

International guidelines are increasingly incorporating recent advances in the management of HER2-mutant NSCLC. The ESMO 2023 consensus on oncogene-driven NSCLC recommends T-DXd following platinum-based chemotherapy in patients with HER2-mutant disease (ESMO level of evidence II, grade A). In parallel, the National Comprehensive Cancer Network (NCCN) guidelines list multiple HER2-directed options in the second-line setting.

Within the NCCN framework, fam-trastuzumab deruxtecan-nxki and HER2-selective tyrosine kinase inhibitors, including zongertinib and sevabertinib, are categorized as preferred regimens, while T-DM1 is designated as useful in certain circumstances. Importantly, no head-to-head comparative data have demonstrated the superiority of one preferred HER2-directed strategy over another, and the guidelines do not establish a hierarchy among these preferred options.

Accordingly, treatment selection beyond first-line therapy should be individualized based on prior treatments, disease burden, toxicity profiles, central nervous system involvement, and patient-specific factors. At present, in the absence of approved first-line HER2-targeted therapies, standard initial management continues to rely on platinum-based chemotherapy, with or without immunotherapy, in accordance with established guidelines.

Given the high incidence of central nervous system metastases in HER2-mutant NSCLC, systematic CNS surveillance and management should be incorporated into longitudinal treatment planning, as discussed in the subsequent section. Overall, this algorithm reflects current guideline recommendations and available clinical evidence, while allowing flexibility to accommodate evolving data and individual clinical contexts.

## 8 CNS metastases: management in HER2-driven NSCLC

CNS metastases represent a major clinical challenge in HER2-mutant NSCLC. Available data suggest that approximately 30–50% of patients develop brain metastases during the course of their disease, underscoring the importance of durable intracranial disease control as systemic outcomes improve (127). Historically, limited blood–brain barrier penetration restricted the effectiveness of systemic therapies, necessitating reliance on local approaches such as stereotactic radiosurgery (SRS), whole-brain radiotherapy (WBRT), or surgery. More recently, the development of HER2-selective TKIs and next-generation ADCs has supported a more integrated role for systemic therapies alongside local approaches (128).

### 8.1 Evidence for clinically active agents

Emerging clinical data indicate that HER2-selective TKIs and ADCs can achieve intracranial activity.

Among HER2-selective TKIs, zongertinib has demonstrated CNS efficacy, with an intracranial objective response rate of approximately 41% and a high rate of intracranial disease control in patients with baseline brain metastases (129). The absence of reported treatment-related interstitial lung disease or pneumonitis to date suggests a favorable safety profile that may facilitate sustained dosing in patients with CNS disease. Sevabertinib has shown preliminary signals of CNS activity, including disease control in patients with baseline brain metastases, although mature analyses with predefined intracranial endpoints are not yet available (65).

Supportive evidence for CNS activity with HER2 inhibition is provided by earlier-generation TKIs. In the ZENITH20 study, poziotinib achieved intracranial responses, with an intracranial ORR of approximately 29% and a median CNS progression-free survival of around 7 months (57). Although poziotinib is no longer widely used because of tolerability concerns, these findings support the biological plausibility of CNS disease control with HER2-targeted TKIs.

Despite their larger molecular size, ADCs can also demonstrate intracranial activity in the setting of brain metastases. In a pooled secondary analysis of the DESTINY-Lung studies, trastuzumab deruxtecan (T-DXd) achieved an intracranial ORR of approximately 50% at the approved 5.4 mg/kg dose, including confirmed complete intracranial responses, while earlier cohorts treated with 6.4 mg/kg showed intracranial ORRs of approximately 30%, with a median CNS response duration of 7–9 months (132) (75). Consistent intracranial activity reported in the breast cancer literature further supports the potential of T-DXd to control CNS disease across HER2-driven malignancies.

Reflecting this evolving evidence base, NCCN guidelines recognize trastuzumab deruxtecan as a systemic treatment option that may be considered for patients with HER2-mutant NSCLC and brain metastases, emphasizing individualized treatment decisions based on disease burden and prior therapies.

## 9 Conclusion and future perspectives

HER2-targeted therapy in non–small-cell lung cancer has rapidly evolved from an area of unmet clinical need to a field with multiple effective treatment options. The development of HER2-selective tyrosine kinase inhibitors and antibody–drug conjugates has substantially improved outcomes for patients with HER2-mutant NSCLC and established HER2 as a clinically actionable oncogenic driver.

Despite these advances, several challenges remain. The optimal sequencing of HER2-targeted agents has not yet been defined, and prospective data are needed to clarify how best to integrate TKIs, ADCs, and other systemic therapies across treatment lines. Acquired resistance is expected to become increasingly relevant as patient survival improves, underscoring the importance of understanding resistance mechanisms and developing rational strategies to overcome them. In parallel, refinement of predictive biomarkers beyond HER2 mutation status may help better individualize treatment selection and avoid unnecessary toxicity.

Future research will focus on optimizing treatment sequencing, exploring combination strategies, and addressing central nervous system disease, which remains a major clinical concern. Ongoing and planned clinical trials will be critical in determining how to sustain and extend the benefits of HER2-targeted therapy.

In summary, the therapeutic landscape for HER2-driven NSCLC has changed substantially, offering new opportunities for durable disease control. Continued clinical investigation and careful integration of emerging evidence into practice will be essential to further improve outcomes for this patient population.
